# The challenge of HIV treatment in an era of polypharmacy

**DOI:** 10.1002/jia2.25449

**Published:** 2020-02-03

**Authors:** David Back, Catia Marzolini

**Affiliations:** ^1^ Department of Molecular and Clinical Pharmacology University of Liverpool Liverpool United Kingdom; ^2^ Division of Infectious Diseases and Hospital Epidemiology Departments of Medicine and Clinical Research University Hospital of Basel and University of Basel Basel Switzerland

**Keywords:** HIV, ageing, comorbidities, polypharmacy, drug‐drug interactions, prescribing issues

## Abstract

**Introduction:**

The availability of potent antiretroviral therapy has transformed HIV infection into a chronic disease such that people living with HIV (PLWH) have a near normal life expectancy. However, there are continuing challenges in managing HIV infection, particularly in older patients, who often experience age‐related comorbidities resulting in complex polypharmacy and an increased risk for drug‐drug interactions. Furthermore, age‐related physiological changes may affect the pharmacokinetics and pharmacodynamics of both antiretrovirals and comedications thereby predisposing elderly to adverse drug reactions. This review provides an overview of the therapeutic challenges when treating elderly PLWH (i.e. >65 years). Particular emphasis is placed on drug‐drug interactions and other common prescribing issues (i.e. inappropriate drug use, prescribing cascade, drug‐disease interaction) encountered in elderly PLWH.

**Discussion:**

Prescribing issues are common in elderly PLWH due to the presence of age‐related comorbidities, organ dysfunction and physiological changes leading to a higher risk for drug‐drug interactions, drugs dosage errors and inappropriate drug use.

**Conclusions:**

The high prevalence of prescribing issues in elderly PLWH highlights the need for ongoing education on prescribing principles and the optimal management of individual patients. The knowledge of adverse health outcomes associated with polypharmacy and inappropriate prescribing should ensure that there are interventions to prevent harm including medication reconciliation, medication review and medication prioritization according to the risks/benefits for each patient.

## 1 Introduction

Effective antiretroviral treatments mean that persons living with HIV (PLWH) have a chronic disease with a life expectancy close to the general population 1, 2, 3, although there are differences in estimates when considering parameters such as HIV transmission risk group, lifestyle, race, gender or CD4 cell counts at treatment initiation 4. Older PLWH includes patients infected at an older age as well as patients diagnosed previously and who are ageing with HIV infection 5. Several modelling studies 6, 7 have projected the increase in median age of patients on antiretroviral treatment over the next decade. Forty percent of the HIV population will be constituted of PLWH aged ≥60 years of whom 28% are predicted to have ≥3 comorbidities 6.

However, age‐related comorbidities result in complex polypharmacy and an increased risk for drug‐drug interactions (DDIs). Furthermore, physiological changes related to ageing may affect pharmacokinetics and pharmacodynamics thereby putting elderly PLWH at risk of inappropriate prescribing. It should be noted that according to the World Health Organization 8, the term elderly refers to ≥65 years old.

Ageing leads to physiological, anatomical and biological modifications that can alter drug pharmacokinetics 9, 10. These changes include a reduced gastric acid secretion and a delayed gastric emptying time, although the clinical relevance remains unclear 10. Drug distribution may be impaired by the reduction in total body water and lean body mass with a relative increase in body fat and an increased distribution of lipophilic drugs. In addition, decreased serum albumin leads to an increase in unbound drug and uptake in peripheral tissue. The observed decrease in hepatic clearance (30% to 40%) in older age results from the decline in both liver mass and blood flow rather than changes in the activity of hepatic enzymes 11. Liver mass reduces by 10% to 15% and by 20% per age decade after the age of 65 years in women and men respectively 10. For many drugs, the most apparent effect of ageing is the progressive decrease in renal clearance explained by a lower glomerular filtration rate 10 resulting in a reduced clearance of renally eliminated drugs. Since elderly individuals are often excluded from clinical trials, there is a lack of data on the effect of ageing on the pharmacokinetics of antiretroviral drugs. Available data have shown that the exposure of the non‐nucleoside reverse transcriptase inhibitor (NNRTI) efavirenz and the integrase inhibitor (INI) raltegravir was not significantly changed in PLWH >60 or 45 to 79 years, whereas plasma concentrations of protease inhibitors (PI) were increased 12, 13, 14. Ageing impacted differently nucleoside reverse transcriptase inhibitors (NRTI)/nucleotide reverse transcriptase inhibitors (NtRTI) as tenofovir exposure was reduced by 8% to 13%, whereas conversely emtricitabine was increased by 19% to 73% in PLWH ≥55 years 15. Dolutegravir maximal concentrations were shown to be increased by 25% in PLWH ≥60 years, however, this change did not modify sleep or daytime functioning 16. There is a need for more pharmacokinetic data in elderly PLWH, especially those with comorbidities or frailty.

The pharmacodynamics can also be impacted by age‐associated physiological changes leading in a more or less pronounced drug effect, particularly for cardiovascular or central nervous systems drugs. The modification of the pharmacodynamic effect is driven by changes in the affinity to receptor sites or in their number as well as the alteration of homeostatic processes with advanced age 17. One important example relates to the reduction in cholinergic receptors in the brain which means that elderly PLWH are more likely to experience central anticholinergic adverse reactions (i.e. cognitive impairment, delirium) therefore drugs with anticholinergic properties should be avoided 18.

Altogether, the presence of comorbidities and age‐related physiological changes predispose elderly PLWH not only to the well‐known risk of DDI with antiretroviral drugs but also to other prescribing issues as discussed in the following sections.

## 2 Discussion

### 2.1 Comorbidities among HIV‐positive individuals

As summarized in Table 1, commonly observed comorbidities which may contribute to the issue of polypharmacy in ageing PLWH are hypertension, dyslipidaemia, diabetes mellitus, kidney disease, cardiovascular disease, respiratory disorders, bone disorders or cancer. Of interest, several studies have reported a higher prevalence of comorbidities in PLWH compared to age‐matched uninfected individuals 5, 19, 20, 21, 22, 23, 24, 25, 26, 27. Furthermore, multimorbidity defined by the concurrent presence of ≥2 comorbidities has been shown to be significantly higher in PLWH compared to uninfected controls, particularly in those with a long history of HIV infection 5, 19, 25.

**Table 1:** Prevalence of comorbidities in ageing PLWH (when available comorbidities in age‐matched uninfected individuals are presented in italic)

Country data source	Population	Mean age, years	Diabetes, %	Dyslipidaemia, %	Hypertension, %	Renal disease, %	CVD disease, %	Bone disorder, %	Respiratory disorder, %	Cancer, %
Brazil 28	451 PLWH	58	14.9	26.7				6.7		3.1
3 HIV centres										
Brazil 29	208 PLWH	57	22.6		62.0	16.8	9.6	52.9		10.6
Brazilian cohort	*208 HIV neg*	*57*	*28.4*		*69.7*	*6.7*	*12.5*	*10.1*		*6.3*
USA 30	2359 PLWH	71	25.9	35.7	47.9		20.9	20.3	31.3	
Medicare	*2 mio HIV neg*	*76*	*24.1*	*46.9*	*59.4*		*19.6*	*21.4*	*26.3*	
Portugal 31	401 PLWH	59	13.5	60.8	39.7	8.0		5.7	9.0	8.0
7 HIV centres										
France 32	16436 PLWH	56	9.1	58.3	21.0	4.5	10.8	6.4		12.3
Dat'AIDS cohort	572 PLWH	78	22.0	60.8	43.5	29.4	23.4	12.6		22.9
France 19	10318 PLWH	56	9.3	23.6	21.0	9.6	9.0			14.6
11 HIV centres										
Europe 33	3797	50 to 60	8.0	79.5	79.0	7.0	7.0			
EuroSIDA cohort^a^	PLWH 1837 PLWH	≥60	17.0	84.0	84.0	23.0	15.5			
Italy 34	965 PLWH	65 to 74	27.5	70.0	60.8	17.1	16.9		6.6	
GEPPO cohort	*224 HIV neg*	*65* to *74*	*22.3*	*57.8*	*66.5*	*5.0*	*18.3*		*9.1*	
	293 PLWH	≥75	31.2	74.6	71.8	26.0	29.2		9.8	
	*91 HIV neg*	≥*75*	*15.4*	*50.0*	*67.0*	*10.0*	*30.8*		*18.9*	
Switzerland 35	2233 PLWH	50 to 64	7.0		69.8					
SHCS cohort	450 PLWH	≥65	16.2		78.9					

CVD, cardiovascular; mio, million.

a Study period 2014.

The earlier occurrence of age‐related comorbidities in PLWH compared to uninfected individuals may be explained by factors such as immune senescence or chronic immune activation 36 as well as lifestyle factors (e.g. smoking, alcohol consumption, recreational drug use), viral coinfections (e.g. hepatitis, sexually transmitted diseases) or toxicity of certain antiretroviral drugs 37. Metabolic disorders 38, 39, renal toxicity 40, or CNS side effects 39 are notably observed with the first‐generation antiretroviral drugs.

As expected, the number of comorbidities in PLWH has been shown to increase with age: 18.4% of PLWH aged ≥75 years from the French Dat'AIDS cohort had ≥4 comorbidities versus 4.3% of those aged 50 to 74 years 32. A similar picture is observed in the Swiss HIV Cohort Study (SHCS) as the number of age‐associated comorbidities is significantly higher in PLWH aged 65 years compared to those aged 50 to 64 years 35. Importantly, the study of the SHCS has demonstrated that the higher number of comorbidities with ageing is correlated with a higher use of comedications and consequently a higher risk to have polypharmacy 35.

Older age, obesity, smoking and duration of HIV infection have been associated with an increased risk for multimorbidity in PLWH 5, 34, 41. Of interest, comorbidities have been shown to co‐occur in the same individual in specific patterns. Furthermore, correlations have also been reported between patterns with, for instance a strong association between cardiovascular and metabolic diseases 42. Finally, comorbidities patterns were shown to have different risk factors with older age and a higher body mass index being risk factors for cardiovascular and metabolic disorders. Finally, comorbidities patterns were shown to affect differently health outcomes with, for example cardiovascular disease being associated with poorer physical health, higher risk of functional impairment, hospitalization and a higher number of medical visits 43. These findings could help the development of targeted interventions to prevent, treat and better manage multimorbidity in PLWH.

### 2.2 Polypharmacy

Polypharmacy is commonly defined as the concurrent administration of ≥5 medications, a cut‐off that has been associated with an increased risk of adverse health outcome 44. In HIV medicine, the term polypharmacy most often refers to non‐HIV medications given in addition to antiretroviral drugs. Polypharmacy has been shown to be common in PLWH aged ≥50 years, ranging from 15% up to 94% as reported by several HIV Cohort analyses summarized in Table 2 34, 45, 46, 47, 48, 49, 50, 51, 52, 53, 54, 55, 56, 57. Of interest, polypharmacy was shown to be less prevalent in the Ugandan cohort possibly due to limited access to care and/or medications 56. This assumption is supported by the observation that access to care, through medication insurance coverage and healthcare use, was shown to be a key driver for polypharmacy in the MACS cohort 57. Large cohort studies comparing the prevalence of polypharmacy in PLWH and age‐matched uninfected individuals have shown higher prevalence in infected individuals across different age categories 52, 58. Of interest, differences in the prevalence of polypharmacy between PLWH and age‐matched uninfected individuals were shown to be less pronounced in older age groups (i.e. 65 to 74 and ≥75 years) 30, 52. This finding may be explained by the natural occurrence of age‐related chronic diseases regardless of HIV infection.

**Table 2:** Prevalence of polypharmacy (≥5 non‐HIV drugs) in PLWH aged 50 years and older

Country	Number PLWH	Age, years	Polypharmacy, %	Reference
Switzerland	111	≥75	60	Livio et al. 2018 51
Switzerland	131	≥65	46	Courlet et al. 2019 45
Italy	1258	≥65	37	Guaraldi et al. 2018 34
USA	1311	≥65	43	Justice et al. 2018 49
USA	89	≥60	74	Greene et al. 2014 46
USA	1715	≥50	36	Ware et al. 2019 57
UK/Ireland	698	≥50	30	Halloran et al. 2019 47
Spain	10073	≥50	47	Lopez‐Centeno et al. 2019 52
Spain	242	≥50	48	Nunez‐Nunez et al. 2018 54
USA	248	≥50	94	Mc Nicholl et al. 2017 53
USA	1312	≥50	54	Holtzman et al. 2013 48
Canada	386	≥50	43	Krentz et al. 2016 50
Japan	526	≥50	35	Ruzicka et al. 2018 55
Uganda	411	≥50	15	Ssonko et al. 2018 56

Medications implicated in polypharmacy belong mostly to drug classes used in older individuals such as cardiovascular drugs, gastro‐intestinal agents, hormone replacement therapies or antiplatelet/anticoagulant medications 52, 59.

Polypharmacy brings several challenges. The related increase in pill burden can have a negative effect of treatment adherence. Although available studies have shown inconsistent findings 60, 61, 62, 63, this is likely explained by the fact that adherence is a complex behaviour involving drug‐related but also psychological factors. Polypharmacy may increase the risk of adverse drug reactions due to the use of medications with overlapping side effects, which may convert asymptomatic side effects to a reason for hospitalization. Polypharmacy has been associated with several adverse health outcomes including physical decline, cognitive impairment, falls, hospitalization and mortality 64, 65, 66, 67, 68, 69. However, it should be highlighted that the causality between polypharmacy and the aforementioned outcomes is difficult to ascertain as the outcomes could be a direct consequence of the primary conditions. Finally, polypharmacy has been associated with an increased risk of DDIs and other prescribing issues such as inappropriate drug use, prescribing cascade or drug‐disease interactions 46, 48, 49, 51, 53, 59.

Inappropriate drugs for use in elderly PLWH are generally defined as drugs for which the risk of an adverse event outweighs the clinical benefit. Therefore, the use of inappropriate drugs can lead to adverse drug reactions with the subsequent risk of starting a prescribing cascade. The latter occurs when an adverse drug reaction is misinterpreted as a new disease leading to the prescription of an unnecessary medication, which in turn, can cause an adverse drug reaction leading to the prescription of more drugs 70. The risk of starting a prescribing cascade is higher in elderly PLWH because they are often polymedicated and because they are more susceptible to adverse drug reactions due to age‐related physiological changes affecting pharmacokinetics, pharmacodynamics and homeostatic processes. Ageing PLWH could also be more susceptible to adverse drug reactions due to long‐term exposure to antiretroviral drugs leading potentially to cumulative toxicity. Finally, elderly have more comorbidities and therefore are at higher risk for drug‐disease interactions. The latter occurs when the prescription of a medication to treat a given condition may adversely aggravate a coexisting condition.

Very few studies have assessed the extent of inappropriate prescribing in elderly PLWH. To date, only three studies have addressed this question using tools such as the Beers criteria and/or the STOPP/START criteria for the detection of inappropriate dosing, indication, treatment duration, drugs or treatment omission 71, 72. These studies have revealed that inappropriate prescribing was common with 52% up to 69% of elderly PLWH presenting at least one medication problem 46, 51, 53. Importantly, prescribing issues included more often non‐HIV medications and other prescribing issues than DDIs with antiretroviral drugs suggesting the need for education on geriatric medicine principles. In addition, there is a need for more real‐world studies that quantify the risk associated with various forms of polypharmacy and DDIs to help guide treatment management.

Interventions to prevent unnecessary polypharmacy and limit inappropriate prescribing include medication reconciliation, medication review and medication prioritization. The decision to prescribe should take into account the risk/benefit of each medication, the care goals, the remaining life expectancy and the current level of functioning as well as the patient preference (patient‐centred approach) 73. In this context, the concept of deprescribing or the process of dose reduction or stopping medications that may be causing harm or no longer provide benefit has gained increasing attention as a means to reduce unnecessary/inappropriate polypharmacy in elderly individuals 74.

Of interest, markers of disease severity like the VACS index developed for PLWH 75 or the Charlson comorbidity index 76 might be useful indices to consider for medication prioritization. Furthermore, these indices may help identify individuals at greatest risk of harm from polypharmacy as they reflect physiological frailty more precisely than age alone.

### 2.3 Evaluating the drug‐drug interaction potential of a drug

The potential for DDIs is mainly investigated before the marketing of a drug. It is important to gain an in‐depth understanding of the disposition of the drug from *in vitro* data, studies in selected animal species (bearing in mind marked species differences in drug handling) and then first in human studies. Some key considerations are metabolic pathways, transporter involvement and protein binding since this will give a framework for understanding the potential of the drug to be a “victim” of DDIs. Similarly, there needs to be early data on the drug as a “perpetrator” of DDIs either by induction or inhibition of metabolic enzymes and/or transporters ‐ these being the key, although not exclusive, pathways of pharmacokinetic interactions. The aim of DDI studies performed on a drug in development is to gain knowledge of how this new chemical entity affects the safety and efficacy of other drugs and vice versa. Specific DDI studies performed in healthy volunteers will be based on plausible interaction mechanisms and key/frequently used medication in the target patient population. Overall, an early understanding of the DDI potential of a drug is critical to ensure safety during clinical phase II and III studies, as well as post approval. Additional studies may be required post‐approval due to emerging science or as a result of case reports of suspected DDIs or population pharmacokinetic data from large phase III real‐world studies.

**Figure 1:**
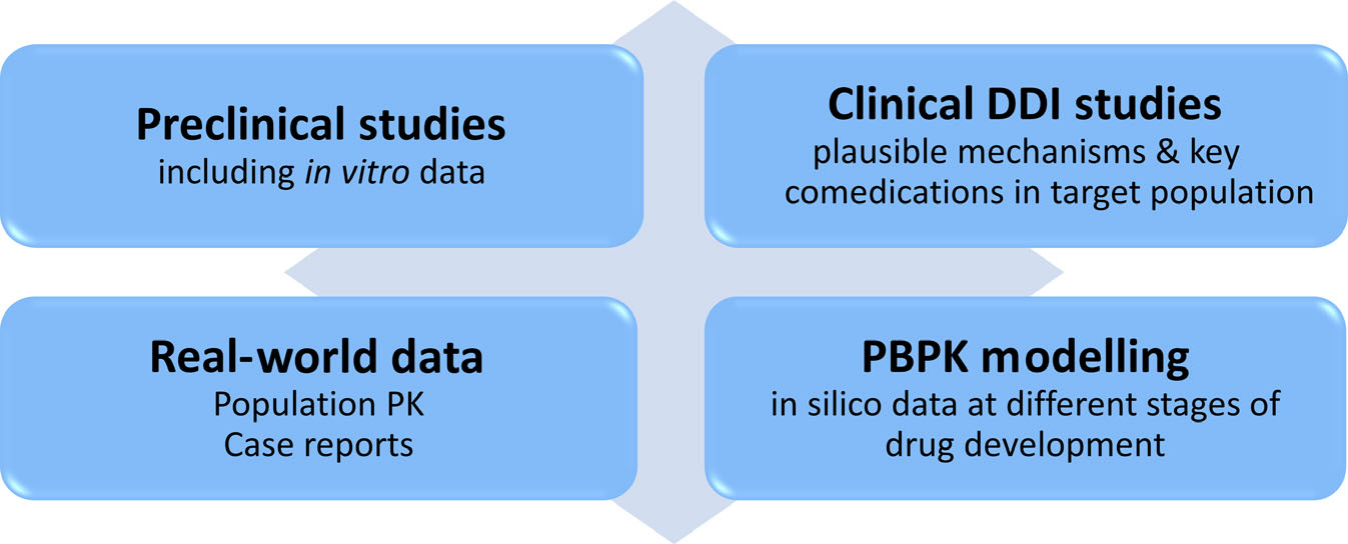
Evaluating the drug‐drug interaction potential of a drug. PBPK, physiologically based pharmacokinetic modelling.

One important emerging area is physiologically based pharmacokinetic modelling (PBPK) which has been applied with significant impact during drug development and post marketing phases and has achieved regulatory acceptance (e.g. FDA, EMA). In brief, PBPK models represent the body and compartments parameterized based on physiology of tissues and organs. PBPK models integrate this physiological description with compound‐specific data to predict the pharmacokinetics of drugs, allowing simulation of the time course of drug concentrations in plasma and tissues. This approach is being increasingly used to simulate and predict DDIs 77, 78. The various approaches to evaluate the DDI potential of a given drug are depicted in Figure 1.

The consequences of an observed or predicted DDI is assessed and treatment recommendations follow. Information about DDIs are presented in the relevant sections of the product label, the purpose of which is to assist the prescriber in the use of a specific medicine. However, a recent assessment of the consistency of DDI information in drug labels in several countries (USA, UK, China, Japan, Korea) showed only a moderate level of agreement among the countries' labelling 79. The study concluded that there is a need for international harmonization of the drug labelling process and regulation to produce standardized information that can ensure safe drug therapy worldwide. To illustrate the point of labelling differences we show the example of the established and other potentially significant DDIs of the integrase inhibitor dolutegravir in the US prescribing information, the European summary of product characteristics and Japan prescribing information 80, 81, 82 (Table 3). It is immediately clear that there is not only different wording used (e.g. for cation‐containing antacids) but different recommendations (e.g. carbamazepine, phenytoin).

**Table 3:** Drug‐drug interactions labelling differences for dolutegravir 80, 81, 82

Comedication	US prescribing information	European summary of product characteristics	Japan prescribing information
Dofelitide	**Contraindicated**	**Contraindicated**	*Pilsicainide* ‐ Caution^a^
Carbamazepine	Same recommendation: DTG 50 mg twice daily in INI naïve patients		
Oxcarbazepine	**Should be avoided**	DTG 50 mg twice daily in INI naïve patients	–a
Phenobarbital	**Should be avoided**	DTG 50 mg twice daily in INI naïve patients	Caution^a^
Phenytoin	**Should be avoided**	DTG 50 mg twice daily in INI naïve patients	Caution^a^
St John's Wort	**Should be avoided**	DTG 50 mg twice daily in INI naïve patients	Caution^a^
Rifampicin	Same recommendation: DTG 50 mg twice daily in INI naïve patients		
Efavirenz	Same recommendation: DTG 50 mg twice daily in INI naïve patients		
Etravirine	Should not be used without ATV/r, DRV/r, or LPV/r	Use 50 mg twice daily without a bPI. Should not be used without bPI in INI‐resistant patients	Use 50 mg twice daily without a bPI. Do not use without either ATV/r, DRV/r, or LPV/r in INI‐resistant patients
Fosamprenavir	DTG 50 mg twice daily in INI naïve patients	No dose adjustment in INI naïve patients or in absence of INI resistance	Do not use in INI‐resistant patients
Cation‐containing antacids	DTG two hours before or six hours after	Antacid two hours after or six hours before	DTG two hours before or six hours after
Iron/calcium supplements	DTG two hours before or six hours after	Antacid two hours after or six hours before	DTG two hours before or six hours after but with food at the same time^a^
Metformin	Close monitoring; limit total daily dose	Dose adjustment should be considered	Administer with care; reduce dose as necessary^a^

ATV/r, atazanavir boosted with ritonavir; bPI, boosted protease inhibitor; DRV/r,darunavir boosted with ritonavir; DTG, dolutegravir; INI, integrase inhibitor; LPV/r, lopinavir boosted with ritonavir.

a Japan label differs from the US prescribing information and the European summary of product characteristics.

While product labels summarize the essential clinical pharmacology and are vital resources, the difference in interpretation of the same DDI data make other resources essential. One seemingly “grey” area is how physicians should use “new” medications in patients on concomitant medications that are not mentioned in the product label. In this context a DDI checker such as that developed by the University of Liverpool (http://www.hiv-druginteractions.org) 83 is an indispensable resource for management of DDIs.

### 2.4 Mechanisms of drug‐drug interactions with antiretroviral drugs

Antiretroviral drugs have a high potential for DDIs as these drugs can be affected by comedications (victim of DDIs) and can also impact comedications (perpetrator of DDIs) resulting in either a lower or higher exposure of the HIV drug or the comedication and consequently to reduced efficacy or toxicity.

**Figure 2:**
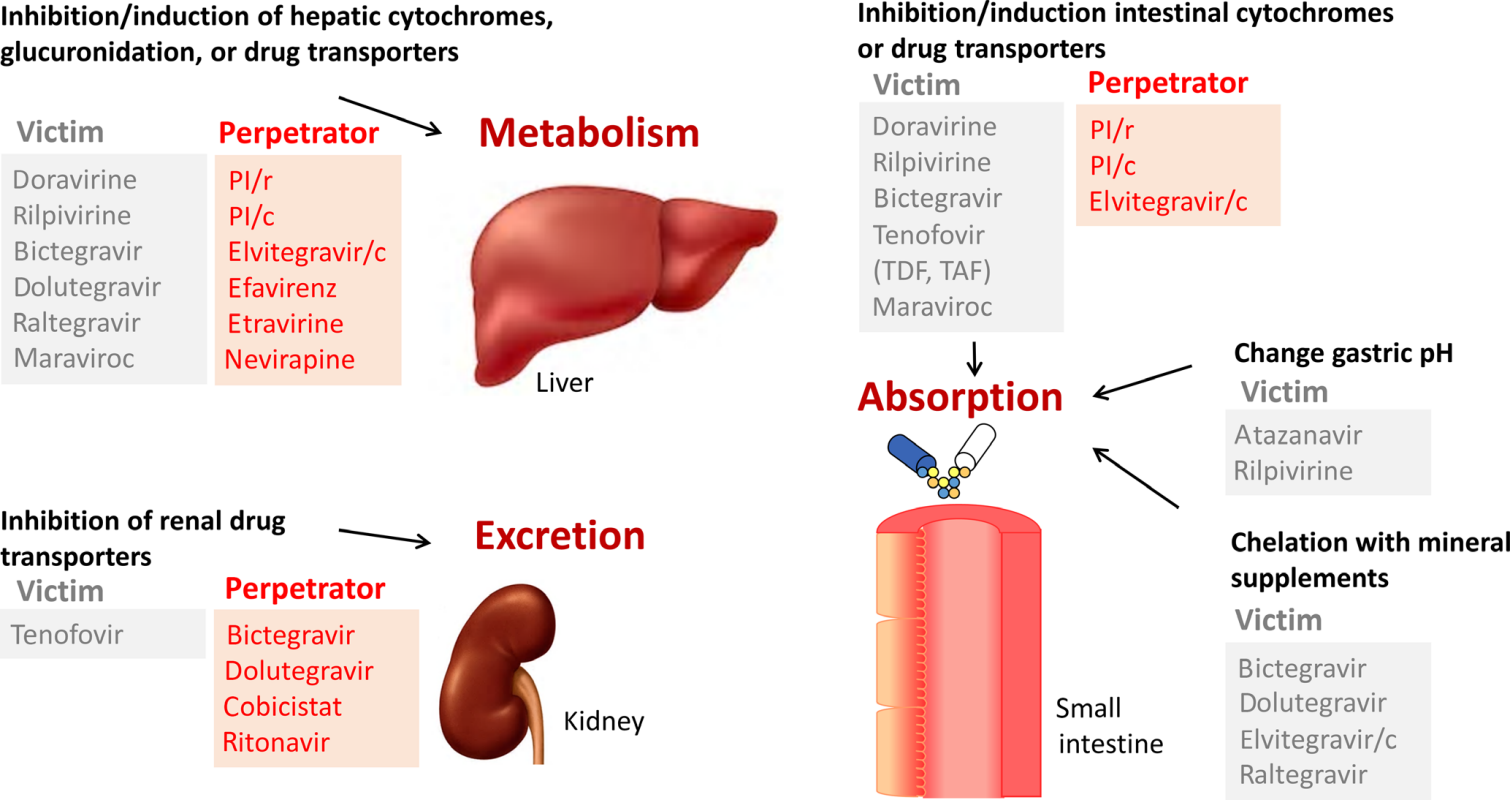
Mechanisms of drug‐drug interactions with antiretroviral drugs. Victim means that the exposure of the antiretroviral drug can be increased or decreased by a comedication with inhibitory or inducing properties on drug‐metabolizing enzymes or drug transporters. Conversely, perpetrator means that the antiretroviral drug inhibits and/or induces drug‐metabolizing enzymes and/or transporters and therefore can alter the exposure of the coadministered drug. Figure reproduced from reference 84 with permission from the journal Taylor & Francis (https://tandfonline.com). c, cobicistat; PI, protease inhibitor; r, ritonavir; TAF, tenofovir alafenamide; TDF, tenofovir disoproxil fumarate.

Pharmacokinetic DDIs with antiretroviral drugs can occur at the level of absorption, metabolism or elimination via the following mechanisms (Figure 2):

‐ *Gastric pH changes*: Antacids markedly decrease the absorption of atazanavir 85 and rilpivirine 86 since a low pH is required for their solubility. There is the potential for efficacy to be compromised.
‐ *Chelation*: Since integrase inhibitors exert their effect by binding to a divalent cation in the active site of the integrase enzyme, the divalent cations aluminium, calcium and magnesium in antacids/supplements, and also iron products are able to form a complex with INIs, thereby impairing their absorption and efficacy 87, 88, 89.
‐ *Inhibition/induction of intestinal cytochrome P450 3A4 (CYP3A4) and/or intestinal transporters*: Rifampicin is a potent enzyme/transporter inducing agent and can affect the oral availability of TAF by increasing P‐gp mediated efflux in the enterocytes. On the other hand, PIs boosted with cobicistat increase the absorption of dabigatran due to the inhibition of intestinal P‐gp. This increases the systemic concentrations of dabigatran and consequently increases the risk of bleeding 90.
‐ *Inhibition/induction of hepatic CYPs and/or glucuronidation enzymes and/or hepatic transporters*: The liver is the major site for DDIs and there are multiple examples of clinically relevant DDIs. An important example is the impact of PIs boosted with ritonavir or cobicistat on the exposure of several statins via inhibition of CYP3A4 and/or hepatic transporters thereby increasing the risk of myopathy or rhabdomyolysis 91. The flip side is induction and there are many clinically relevant inducers of CYP enzymes, glucuronyl transferases and influx (e.g. OATP1B1/3) and efflux (e.g. P‐gp; MRP2) drug transporters.
‐ *Inhibition of renal tubular transporters*: There are some endogenous compounds (e.g. creatinine) and several therapeutic agents (e.g. metformin) which are actively transported in the renal proximal tubule. Dolutegravir and bictegravir inhibit the OCT2 mediated uptake of metformin in the tubular cells, whereas cobicistat and ritonavir inhibit metformin secretion in the urine via the multidrug and toxin extrusion protein MATE1; both mechanisms increase the exposure of the antidiabetic drug 92, 93.

Pharmacodynamic DDIs may be encountered with certain antiretroviral drugs when coadministered with drugs characterized by a similar toxicity profile thereby increasing the risk of additive adverse drug effects. As an example, both acute and chronic renal toxicity have been associated with TDF 40, 94. Thus, coadministration of TDF and nephrotoxic medications can increase the risk of nephrotoxicity, particularly in PLWH with pre‐existing renal impairment or when treatment are administered for a long duration 95. Another example is synergistic QT prolongation when a patient is taking more than one drug with a QT liability. The NNRTI rilpivirine has been associated with prolongation of the QTc interval at supratherapeutic doses and there are other drugs (e.g. escitalopram) which carry warnings of dose‐dependent QT effects.

**Figure 3:**
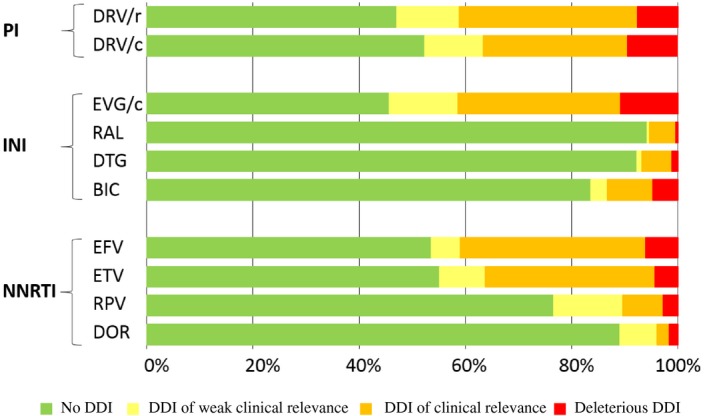
Drug‐drug interaction profiles of selected antiretroviral drugs. Percentage of green, yellow, amber and red DDIs considering 750 comedications listed in the Liverpool HIV interaction website 83 for selected antiretroviral drugs belonging to the protease inhibitor (PI), integrase inhibitor (INI) and non‐nucleoside reverse transcriptase inhibitor (NNRTI) classes. BIC, bictegravir; DRV/c, darunavir boosted with ritonavir; DRV/r, darunavir boosted with ritonavir; DOR, doravirine; DTG, dolutegravir; EVG/c, elvitegravir boosted with cobicistat; EFV, efavirenz; ETV, etravirine; RAL, raltegravir; RPV, rilpivirine.

When possible, antiretroviral drugs with a lower potential for DDIs such as the unboosted INIs (raltegravir, dolutegravir, bictegravir), or the NNRTIs doravirine or rilpvirine should be favoured where there is polypharmacy. Figure 3 shows the DDI profile of antiretroviral drugs based on an evaluation of DDI data with ≥750 comedications taken from the website http://www.hiv-druginteractions.org 83.

Selected clinically important DDIs and their management are presented in Table 4. More information on DDIs can be found in the Liverpool HIV drug interactions website 83.

**Table 4:** Selected clinically important drug‐drug interactions and their management

Drug class	ARV	Comments/recommendations
Statins^a^	Boosted PI Elvitegravir/c	Boosted ARVs increase the exposure of several statins. The magnitude of the DDI depends on the metabolic pathway of the statin and its affinity to hepatic drug transporters 91 Simvastatin, lovastatin: contraindicated due to large magnitude DDI and related risk of rhabdomyolysis Other statins: start with low dose and titrate to effect. ATV is a strong inhibitor of the hepatic uptake transporter OATP1B1 resulting in large magnitude DDIs with statins. Do not exceed 10 mg/day of atorvastatin and rosuvastatin when coadministered with ATV
Calcium channel inhibitors^a^	Boosted PI Elvitegravir/c	Boosted ARVs increase the exposure of calcium channel inhibitors due to inhibition of CYPs and thereby the hypotensive effect Start at a lower dose and titrate based on blood pressure response. Consider a 50% dose reduction for amlodipine and diltiazem 96, 97 Lercanidipine: contraindicated
Antidiabetics^a^	Boosted PI Bictegravir Elvitegravir/c Dolutegravir	Sulfonylureas: boosted ARVs can potentially increase sulfonylureas concentrations due to inhibition of CYPs, monitor glycaemic control and adjust dose as necessary. Metformin: DTG >BIC increase metformin exposure due to inhibition of renal transporter OCT2. Consider adjusting metformin dose when starting DTG. No need to adjust metformin dose in patients treated with BIC and with normal renal function otherwise close monitoring is advised 92, 93 Saxagliptin: maximal daily dose with boosted ARVs: 2.5 mg Dapagliflozin, empagliflozin, exenatide, linagliptin, liraglutide, sitagliptin, vildagliptin: no clinically relevant DDIs
Vitamine K antagonists^a^	Boosted PI Elvitegravir/c	Boosted ARVs have both inhibitory/inducing effects on CYPs and therefore are expected to alter vitamin K antagonists effect. Closely monitor INR 98, 99, 100, 101, 102 Dose adjustments may be needed when switching pharmacokinetic booster as ritonavir has inducing properties on CYPs, whereas cobicistat does not 103
Direct‐acting anticoagulants^a^	Boosted PI Elvitegravir/c	Boosted ARVs cause clinically significant DDIs with direct‐acting anticoagulants due to inhibition of CYPs and/or transporters. Data on management of DDIs are limited 90, 104, 105, 106 Apixaban, rivaroxaban: avoid Dabigatran: coadministration is possible with PI boosted with ritonavir* but is not possible with cobicistat boosting. (*a dose adjustment of dabigatran might be needed in patients with mild or moderate renal insufficiency) Edoxaban: consider a dose reduction from 60 to 30 mg
Antiplatelets^a^	Boosted PI Elvitegravir/c	Aspirin: no DDIs Clopidogrel: boosted ARVs alter antiplatelet effect. Coadministration with boosted ARVs is not possible; use alternative antiplatelet agents or unboosted regimens 107, 108, 109 Prasugrel: boosted ARVs do not alter antiplatelet effect. Coadministration with boosted regimens is possible 109 Ticagrelor: contraindicated as boosted ARVs may substantially increase ticagrelor concentrations and increase the risk of bleeding
Antacids H_2_‐receptor blockers Proton pump inhibitors	Atazanavir Rilpivirine	Solubility of ARV decreases as pH increases 85, 86. Administration recommendations: Antacids: ATV: two hours before or after antacid; RPV: four hours before or two hours after antacid H_2_‐receptor blockers: ATV: simultaneous administration or >10 hours after H_2_‐blocker. The dose of H_2_‐blocker should not exceed the equivalent of 40 mg famotidine twice daily (treatment naïve patients) or the equivalent of 20 mg famotidine twice daily (treatment experienced patients); RPV: four hours before or twelve hours after H_2_‐blocker Proton pump inhibitors: contraindicated
Antacids Mineral supplements (iron, calcium, magnesium)	Bictegravir Dolutegravir Elvitegravir/c Raltegravir	Integrase inhibitors form a complex with divalent cations at the level of the gastro intestinal tract thus reducing their absorption 87, 88, 89, 110. Administration recommendations: BIC: two hours before or six hours after antacids; simultaneous with mineral supplements DTG: two hours before or six hours after antacids or mineral supplements EVG/c: separate by four hours from antacids or mineral supplements RAL: not recommended with aluminium‐ and magnesium‐containing antacids. Coadministration possible with calcium carbonate‐containing antacids but only with RAL twice daily. Separate by four hours from mineral supplements, only administration of RAL twice daily possible
Corticosteroids^a^	Boosted PI Elvitegravir/c	Boosted ARVs inhibit steroids metabolism thereby increasing the risk of Cushing syndrome. Risk is not limited to oral administration but may also occur after topical, ocular, intra‐articular or intrathecal administration of steroids 111, 112, 113. The risk of Cushing syndrome is not eliminated by reducing the dosage of the corticosteroid. Avoid boosted ARVs when possible or, if unavoidable, use a corticosteroid with a lower propensity to cause Cushing syndrome with periodic control of cortisol Budenoside, fluticasone, triamcinolone, mometasone: contraindicated Beclomethasone, methylprednisolone, hydrocortisone: can be used with boosted ARVs Dexamethasone can reduce the exposure of boosted ARVs particularly if used at high doses and for a long duration. Use with caution
Antituberculosis drugs Rifampicin, rifabutin Bedaquiline^a^ Delamanid Ethambutol, isoniazid, linezolid, pyrazinamide	PI/r	Contraindicated with rifampicin, alternative rifabutin 150 mg once daily
	PI/c	Contraindicated with rifampicin, alternative rifabutin 150 mg every other day
	Elvitegravir/c	Contraindicated with rifampicin, alternative rifabutin 150 mg every other day
	Bictegravir	Contraindicated with rifampicin and rifabutin
	Dolutegravir	Dolutegravir 50 mg twice daily with rifampicin, dolutegravir 50 mg once daily with rifabutin [114]
	Raltegravir	Raltegravir 400 mg or 800 mg twice daily with rifampicin [115], raltegravir 400 mg twice daily with rifabutin
	Doravirine	Contraindicated with rifampicin, alternative doravirine 100 mg twice daily with rifabutin
	Etravirine	Contraindicated with rifampicin, alternative rifabutin 300 mg once daily (if etravirine is administered without PI)
	Rilpivirine	Contraindicated with rifampicin and rifabutin
	Efavirenz	Efavirenz 600 mg once daily with rifampicin [116], increase daily dose rifabutin by 50% in presence of efavirenz
	Boosted PI Elvitegravir/c	Boosted ARVs inhibit bedaquiline metabolism resulting in increased exposure and related increased risk of QT interval prolongation. Given bedaquiline's prolonged half‐life, coadministration with boosted ARV should not exceed 14 days. Monitor ECG and transaminases. Coadministration with saquinavir is contraindicated
	Boosted PI Elvitegravir/c	Boosted ARVs can increase delamanid exposure resulting in an increased risk of QT interval prolongation. Monitor ECG. Coadministration with saquinavir is contraindicated
	ARVs	No DDIs

More information on DDIs can be obtained from the University of Liverpool HIV drug interactions website: http://www.hiv-druginteractions.org 83. ARV, antiretroviral drug; ATV, atazanavir; BIC, bictegravir; c, cobicistat; CYP, cytochromes; DDI, drug‐drug interaction; DTG, dolutegravir; EVG/c, elvitegravir/cobicistat; OATP1B1, organic anion transporting polypeptide 1B1; OCT2, organic cation transporter 2; PI, protease inhibitor; PI/c, protease inhibitor boosted with cobicistat; PI/r, protease inhibitor boosted with ritonavir; RAL, raltegravir; RPV, rilpivirine.

a Drug exposure can be lowered when coadministered with the non‐nucleoside reverse transcriptase inhibitors efavirenz, etravirine and nevirapine.

### 2.5 Current issues when managing drug‐drug interactions

One current issue is that only few drug associations are evaluated in clinical studies, thus guidance on how to manage DDIs is mostly theoretical or is lacking, particularly when associating mutually interacting drugs as often encountered in clinical practice. A good knowledge of the metabolic pathway of drugs and a good understanding of the mechanisms of DDIs as well as the therapeutic index are essential to predict the risk of having a clinically relevant interaction. A strong inhibition or induction of a major metabolic pathway is generally expected to cause a large magnitude DDI that may require dosage adjustment. Conversely, the magnitude of DDIs will be mitigated when drugs have multiple metabolic or elimination pathways as metabolism and elimination can still occur through the unaffected pathways 117. A misunderstanding of these concepts and of the different DDI of each antiretroviral drugs can lead to an overestimation of the risk of DDI leading consequently to sub‐optimal treatment. This issue has been reported for antidepressants since a larger proportion of PLWH were shown to have sub‐therapeutic antidepressants levels compared to uninfected individuals suggestive of a deliberate lower dosing as clinicians fear DDIs with antiretroviral drugs 118. It should be highlighted that most antidepressants are metabolized by several cytochromes and therefore the magnitude of DDIs with boosted regimens tend to be mitigated. In addition, the pharmacokinetic boosters ritonavir and cobicistat inhibit only weakly cytochrome 2D6 103, which is the major contributor of the metabolism of most antidepressants. Underestimating the risk of DDIs can also occur; this is notably exemplified with the DDI between boosted antiretroviral drugs and corticosteroids. The coadministration of boosted antiretroviral drugs and potent corticosteroids is contraindicated due to the risk of developing a Cushing syndrome. Nevertheless, these drugs are being used together in clinical practice as indicated by two large independent European cohort studies 52, 119. The fact that corticosteroids are administered by different routes (oral, inhalation, intra‐articular, topical) may lead to an underestimation of the risk of DDI. In addition, corticosteroids are used across a large variety of medical specialties (dermatology, pneumology or rheumatology) and therefore are likely to be prescribed by non‐HIV specialists who are not aware of DDIs with antiretroviral drugs.

### 2.6 Drug‐drug interactions and long‐acting antiretroviral drugs

Long‐acting drugs and formulations are an established part of the management of several medical conditions including contraception, schizophrenia and osteoporosis. Long‐acting drug delivery is considered to be a key solution to the problem of poor adherence and since daily oral pills remain a barrier to long‐term suppression of viral replication in PLWH there is understandably much interest in both injectables and implants of antiretrovirals 120, 121. Long‐acting nanocrystal suspensions of the INI cabotegravir and the NNRTI rilpivirine are in advanced clinical development with data from large Phase III studies in maintenance therapy (ATLAS, FLAIR) recently presented at IAS 2019 122. There are other exciting developments using implant technology with non‐degradable subcutaneous implants of two NRTIs ‐ tenofovir alafenamide (TAF) and 4ʹ‐ethynyl‐2‐fluoro‐2ʹ‐deoxyadenosine (EFdA; MK8591) ‐ about to enter clinical testing.

Given that with non‐oral drug administration, the first pass metabolism is bypassed (i.e. the initial metabolism/disposition within the gastrointestinal tract and liver), should it be anticipated that long‐acting regimens will be substantially devoid of DDIs? Clearly, DDIs are drug specific and all aspects involved in the disposition of a given compound have to be considered. In addition, DDIs data from other therapeutic areas have to be reviewed. Considering contraception, the levonorgestrel (LNG) subdermal implants are a highly efficacious and safe form of long‐acting reversible contraception. However, >50% lower LNG exposure was shown in women receiving the LNG subdermal implant with efavirenz‐based antiretroviral treatment compared to antiretroviral treatment‐naïve women in Uganda 123. Despite doubling the dose of the LNG implants, LNG concentrations remained >30% lower when women were on efavirenz‐based antiretroviral treatment 124. Therefore, there is uncertain contraceptive effectiveness even when modifying the dose to overcome the DDI.

Going forward with long‐acting antiretrovirals, it is almost certain that it will be data from short‐term oral DDIs studies that will help inform PBPK modelling. For example rifampicin administration was shown to reduce the exposure of single oral dose cabotegravir by 59% 125. Rajoli et al 77 then designed PBPK models (verified against the observed data for oral cabotegravir, rilpivirine and rifampicin) to predict the DDI of the long‐acting regimen. According to the models, there was a predicted reduction in cabotegravir exposure of 41% and rilpivirine of 82% for the first maintenance dose with 600 mg once daily oral rifampicin. This strongly suggests that coadministration of rifampicin with these long‐acting formulations will be problematic and strategies will need to be considered to overcome the DDI. However, increasing the dose is unlikely due to volume of injection and shortening the dosing interval will present logistical challenges. Therefore, there are some important challenges relating to DDIs when considering the role out of long‐acting regimens.

## 3 Conclusions

DDIs in HIV really came to the forefront of attention more than 20 years ago with the PI era and the realization that boosting of the PI often also resulted in the boosting of other comedications. Although we have moved into the INI era and have unboosted regimens with a greatly reduced liability to DDIs, there still needs to be an awareness of relevant DDIs both with INI and the earlier generation antiretroviral drugs which are still important in certain settings. This is particularly relevant in the older population who often have multiple comorbidities and therefore polypharmacy. DDIs are still an issue we have to face and manage.

Strategies to prevent prescribing errors are important which must include education on key DDIs with each class of antiretroviral drugs and on prescribing principles for specific groups of patients. Medication reconciliation and regular medication review is essential with de‐prescribing if appropriate.

As we look to the future, there are clearly exciting developments in antiretroviral therapy particularly in relation to long‐acting injectables and implants. The question then is “will DDIs still be an issue”? Despite bypassing gastrointestinal absorption there are still hepatic DDIs to consider (and maybe interactions relating to the injection or implant site) and so we need to be clear on the process of generating key data (likely PBPK modelling) and the strategies to deal with clinically relevant DDIs.

## Competing interests

DB has received educational grants for http://www.hiv-druginteractions.org from Gilead, MSD, Janssen, ViiV. Honoraria for speakers' bureau or advisory boards received from Gilead, MSD, ViiV. CM received speaker honoraria for her institution from MSD.

## Authors' contributions

David Back and Catia Marzolini designed the review, drafted the manuscript and critically revised it.
